# Male Parameatal Urethral Cyst: A Report of Two Cases and Literature Review

**DOI:** 10.1155/criu/5555012

**Published:** 2026-04-18

**Authors:** Loukas Charalambous, Joshua Luzuriaga, Michael Ernst

**Affiliations:** ^1^ Department of Urology, Aristotle University of Thessaloniki School of Medicine, Thessaloniki, Greece, auth.gr; ^2^ Department of Pathology, Stony Brook University Hospital, Stony Brook, New York, USA, stonybrookmedicine.edu; ^3^ Department of Urology, Stony Brook University Hospital, Stony Brook, New York, USA, stonybrookmedicine.edu

## Abstract

**Background:**

Parameatal urethral cysts are rare, benign cystic lesions at the urethral meatus, most often occurring in females during childhood. Fewer than 100 cases have been documented, and their pathogenesis remains unclear. This case series describes the symptoms, histological characteristics, and postoperative outcomes of two adolescent males with parameatal urethral cysts and provides an updated review of the literature.

**Case Presentation:**

Two male adolescents (Ages 16 and 17) without prior medical history presented with cystic lesions at the ventral urethral meatus. One reported mild voiding discomfort and altered urinary stream, while the other experienced discomfort and intermittent pain. Both described gradual cyst enlargement over several years. Examination revealed clear, fluid‐filled cysts measuring 1–2 cm. Each underwent complete cyst excision. Pathology demonstrated a urothelial‐type cyst in one case and a mixed‐type cyst in the other.

**Results:**

Both patients demonstrated excellent postoperative cosmesis and complete symptom resolution at 1‐month follow‐up.

**Conclusion:**

Parameatal urethral cysts are rare benign lesions of uncertain origin. Patients typically present due to discomfort or cosmetic concerns. Complete surgical excision remains the gold standard, offering excellent cosmetic and functional outcomes with minimal recurrence risk. These cases contribute to the limited literature and reinforce excision as the preferred treatment.

## 1. Introduction

Parameatal urethral cysts are rare congenital or acquired benign cysts, typically < 1 cm and located on the lateral or ventral margin of the urethral meatus [1, 2]. First described by Ohno in 1919, they have since been reported only sparingly (Thompson and Lantin 1956, Shiraki 1975, Hill et al. 1977) [3]. Fewer than 100 cases have been documented [4]. They are more common in males and usually present before puberty, with reported median ages of 34–41 months [5, 6]. Their etiology remains uncertain [7]. Although often asymptomatic, patients may experience urinary retention, dysuria, altered urinary stream, and dyspareunia, with symptoms correlating with cyst size. Complete surgical excision remains the treatment of choice [4].

## 2. Case Presentation

### 2.1. Case 1

**Figure 1:**
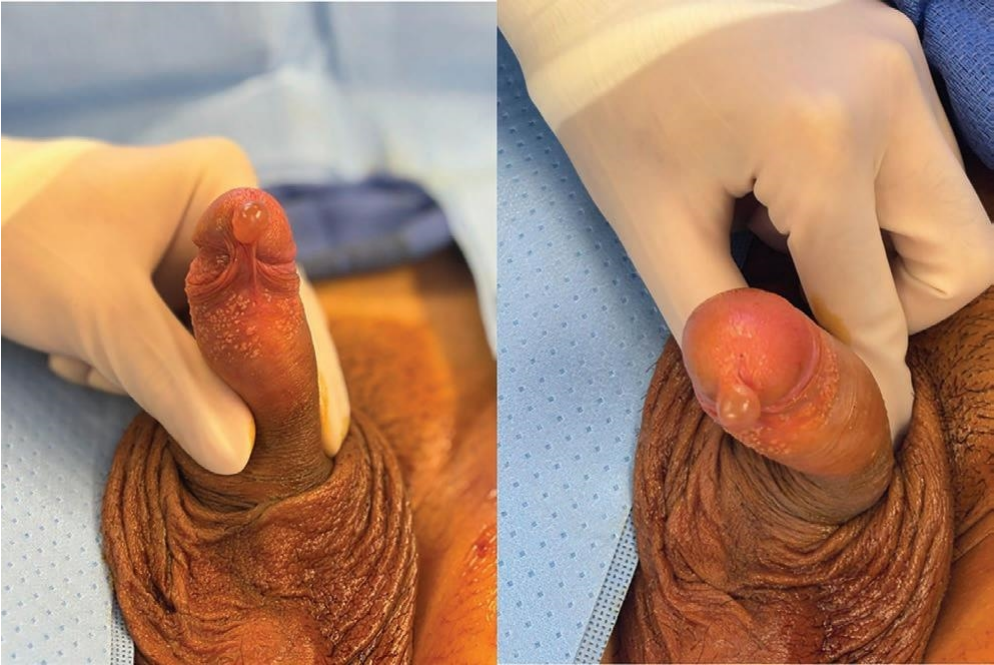
Clinical appearance of parameatal urethral cyst.

A 17‐year‐old Hispanic male with no significant past medical history presented with a painless cystic mass on the glans penis. He reported a 6‐year history of gradual enlargement. His primary concern was cosmesis, though he noted mild voiding discomfort and a change in urinary stream. He denied trauma, STIs, or family history of similar lesions. Examination revealed a clear, fluid‐filled 2‐cm cyst at the ventral meatus (Figure 1).

**Figure 2:**
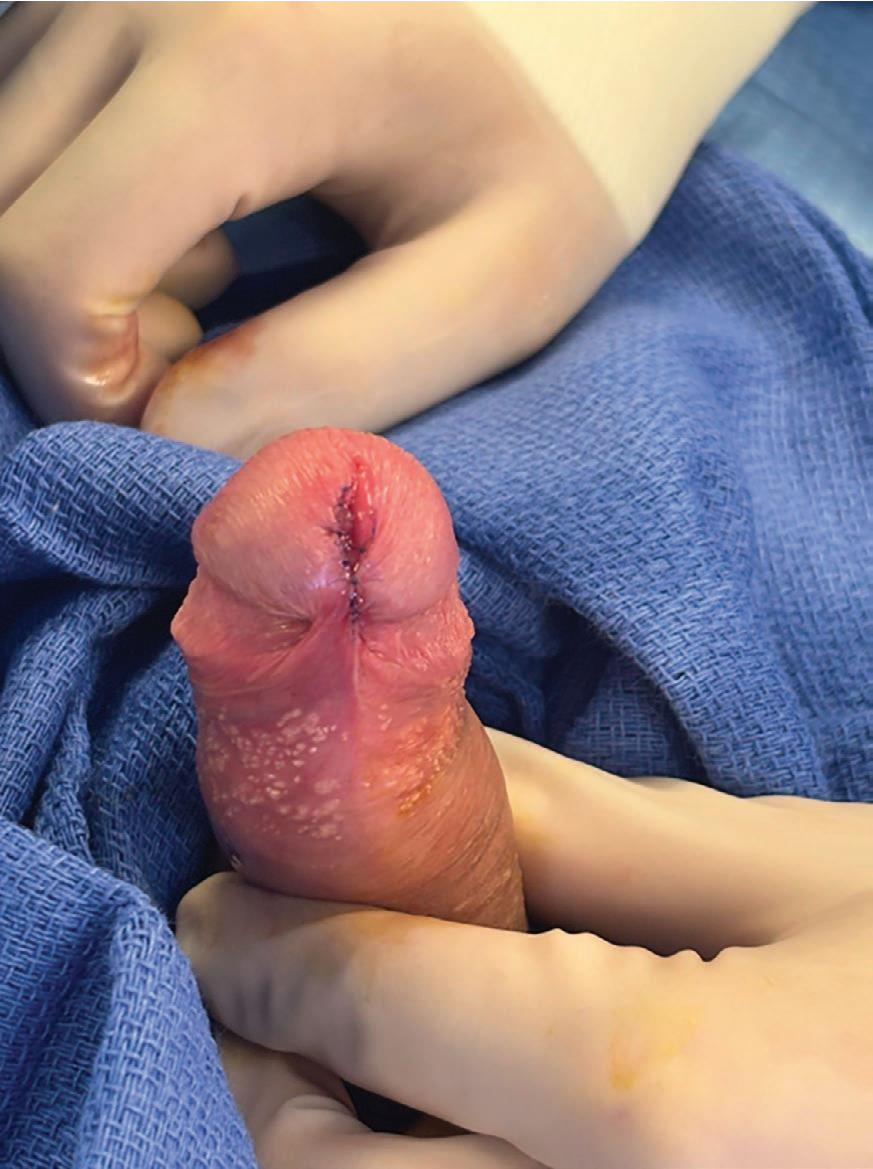
Intraoperative appearance after excision and closure.

**Figure 3:**
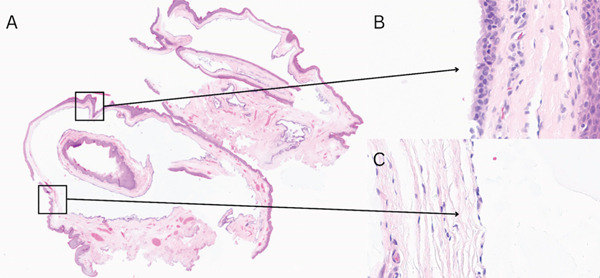
Microscopic appearance of the urothelial‐type cyst (H&E): (A) ×1; (B, C) ×40.

**Figure 4:**
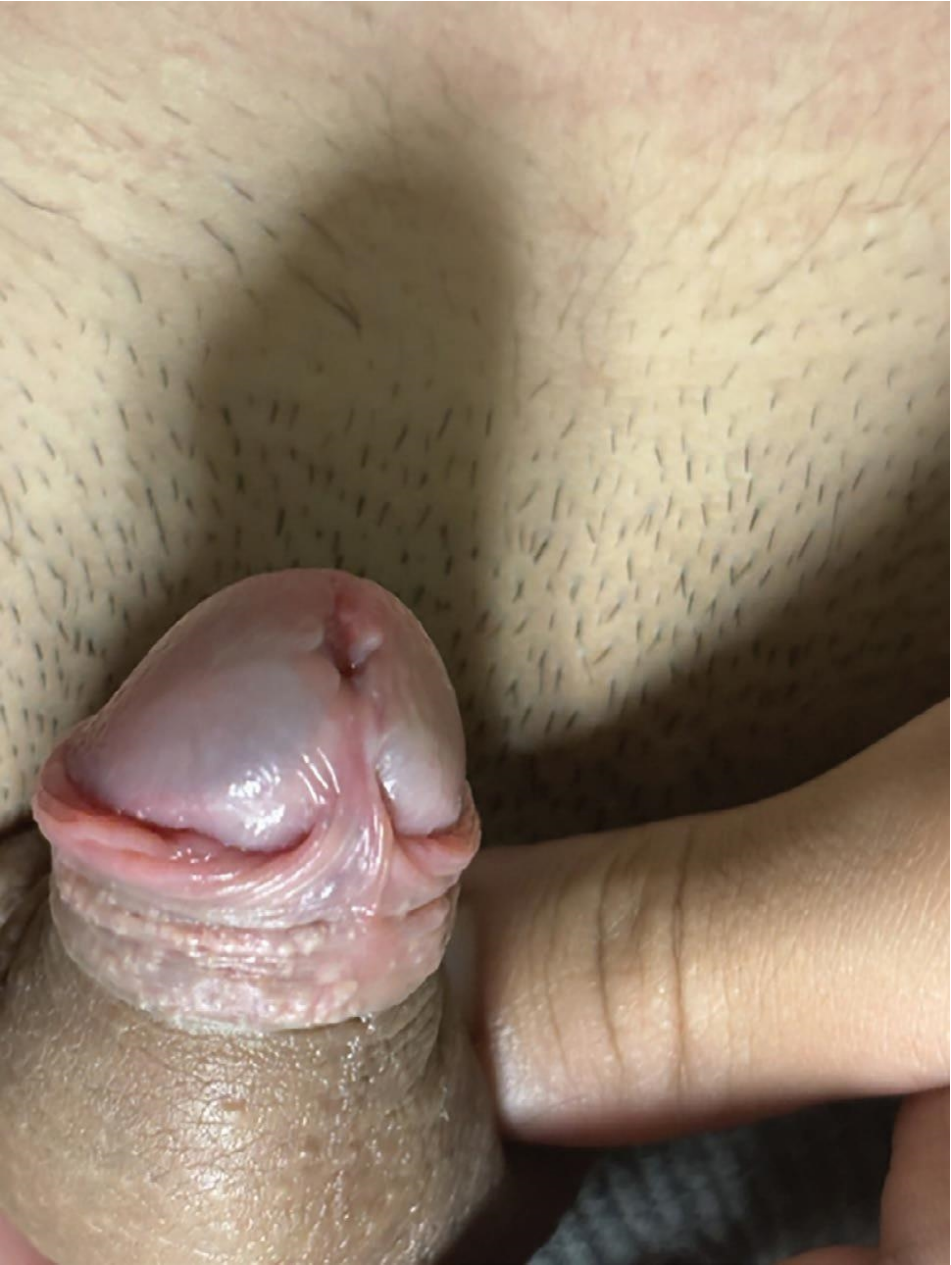
Postoperative appearance.

The patient underwent complete excision under sedation. A circumferential incision was made around the cyst, which was removed in its entirety. The defect was closed with interrupted absorbable sutures, and bacitracin was applied. Intraoperative findings are shown in Figure 2. Pathology demonstrated a cystic wall lined by multilayered cuboidal epithelium, consistent with a urothelial‐type cyst (Figure 3). At 1‐month follow‐up, cosmesis was excellent, and urinary symptoms had resolved (Figure 4).

### 2.2. Case 2

**Figure 5:**
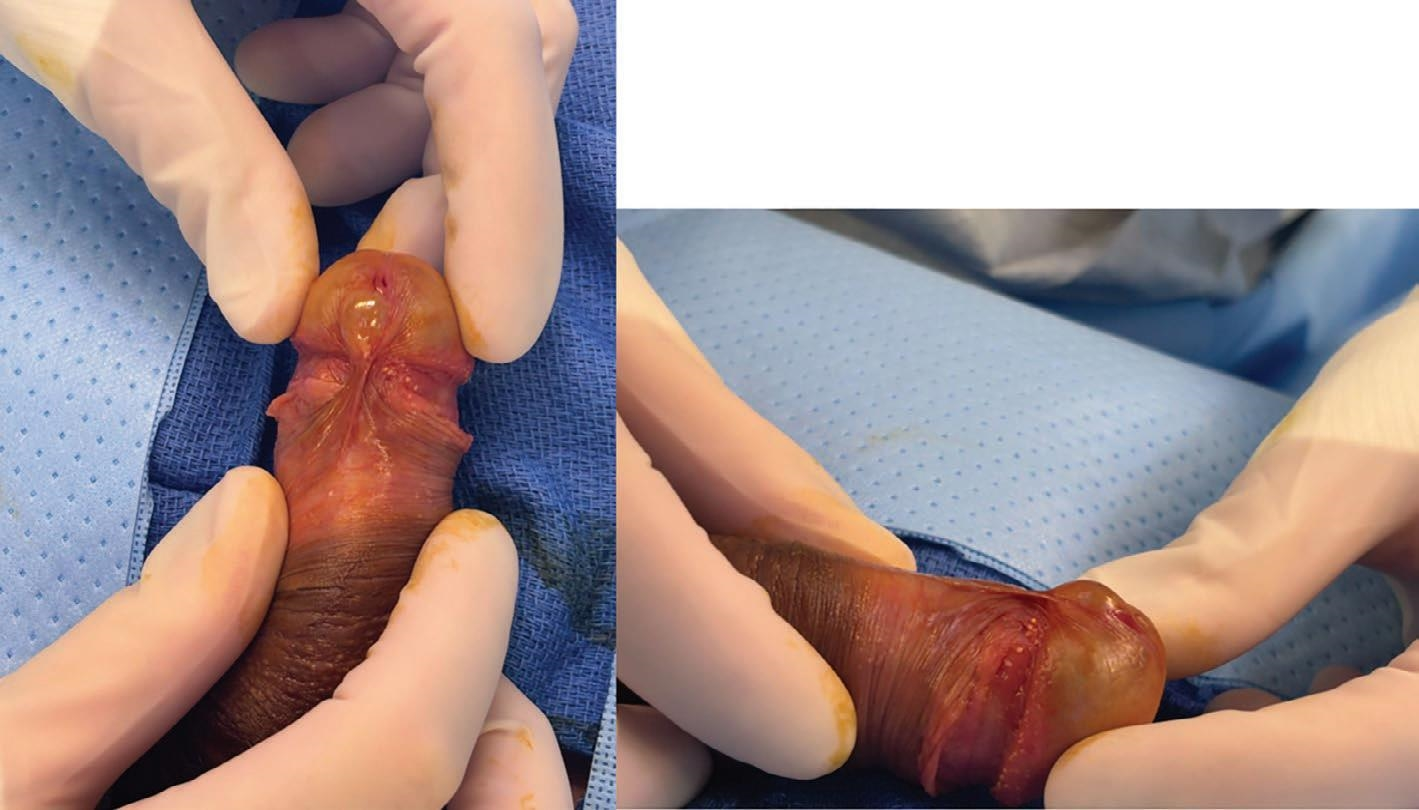
Clinical appearance of parameatal urethral cyst.

**Figure 6:**
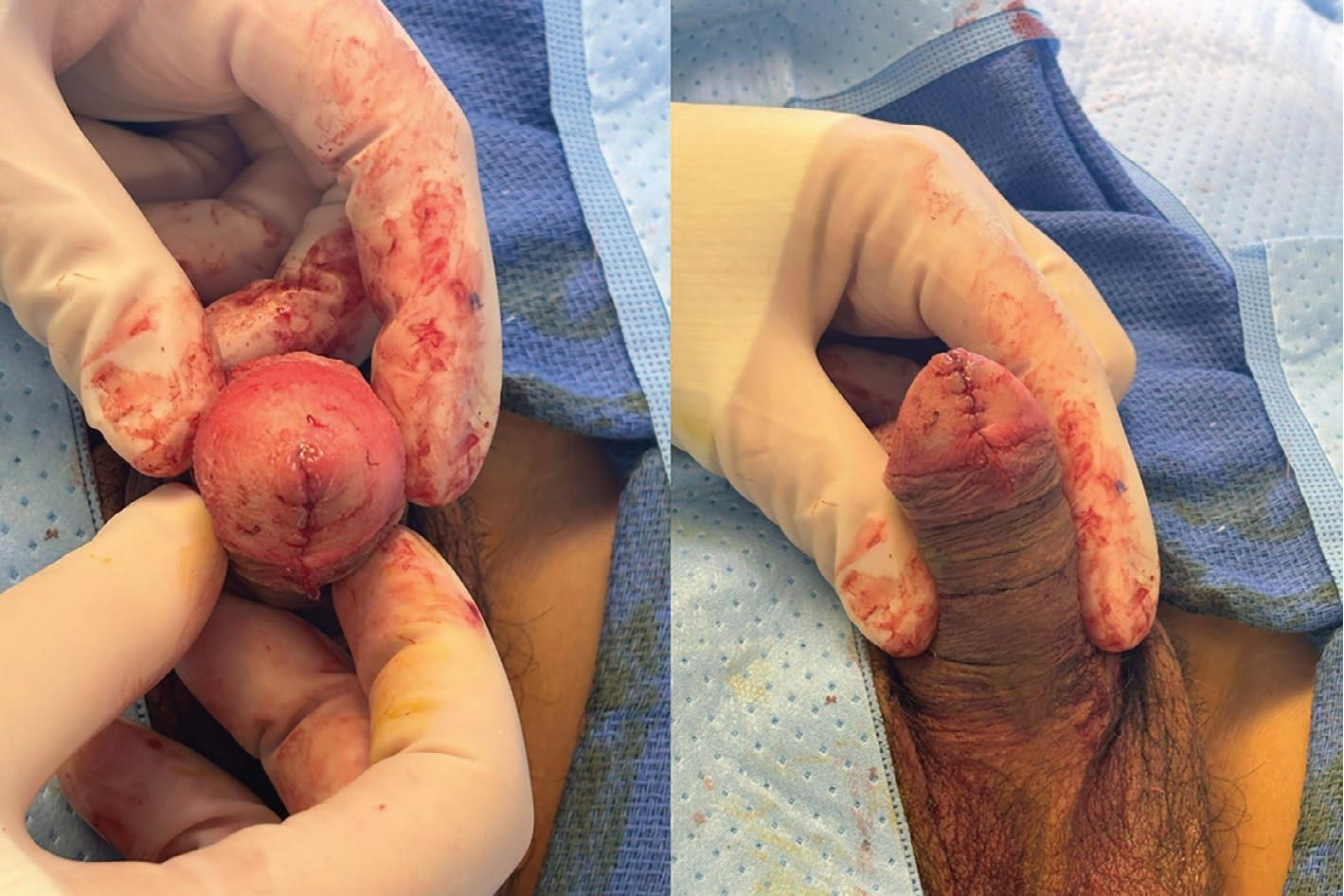
Intraoperative appearance after excision and closure.

**Figure 7:**
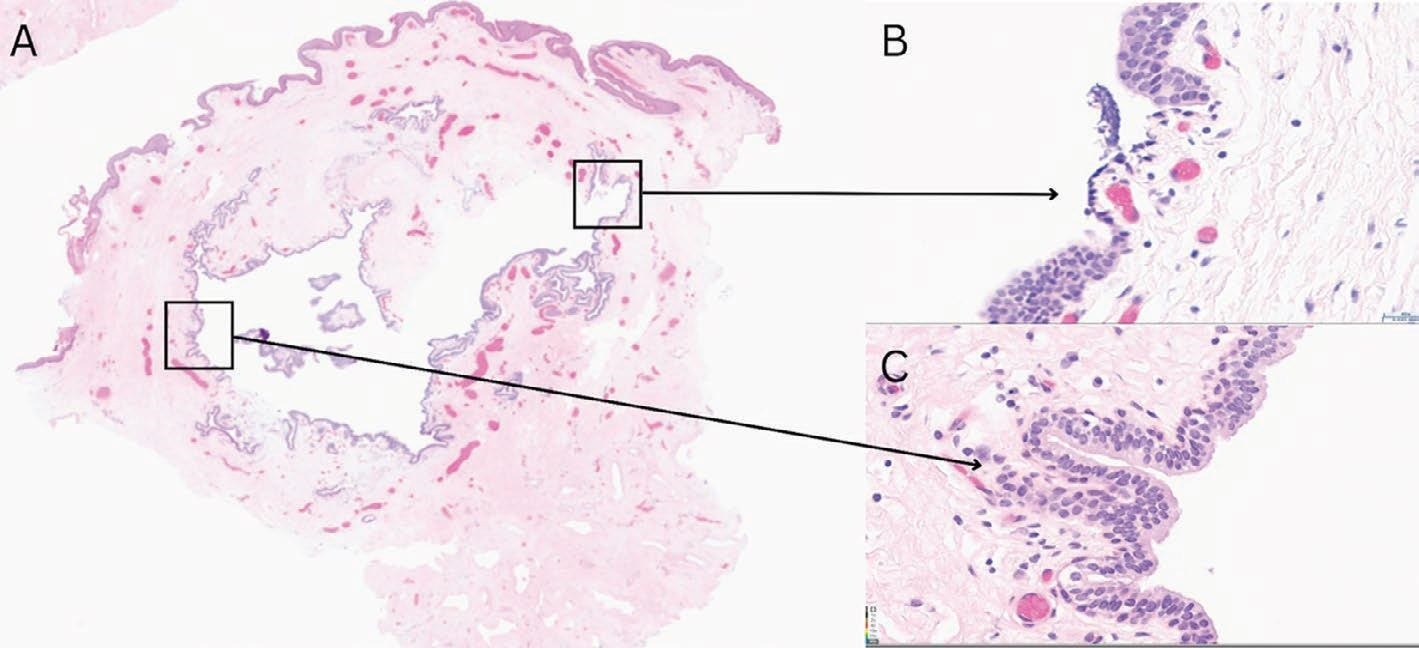
Microscopic appearance of the mixed‐type cyst (H&E): (A) ×1; (B, C) ×40.

A 16‐year‐old Hispanic male presented with a 1‐cm ventral glans cyst associated with 3 years of enlargement, discomfort, and intermittent pain. He denied dysuria, stream changes, UTIs, trauma, or family history of similar findings. Examination revealed a mildly tender, fluid‐filled cyst abutting the meatus (Figure 5). The cyst was excised under sedation (Figure 6). Histopathology demonstrated columnar and urothelial cell lining, consistent with a mixed‐type cyst (Figure 7). At 1‐month follow‐up, cosmetic appearance was excellent with no recurrence.

## 3. Discussion

Parameatal urethral cysts typically arise on the lateral or ventral aspect of the meatus and rarely present bilaterally. Most measure around 1 cm and may enlarge over time [1]. Both cases here demonstrated slowly enlarging ventral lesions measuring 1–2 cm.

Multiple diagnostic terms have been used, including penile mucoid cyst, hidrocystoma, apocrine cystadenoma, and urethromucoid cyst [7, 8]. Their etiology is debated. Thompson and Lantin suggested persistence of cystic spaces during delamination of the foreskin from the glans. Shiraki proposed paraurethral duct obstruction, later supported by Oka et al. and Yoshida et al. [9–11]. Hill and Ashken speculated infection as a contributing factor, though this remains disputed [12, 13].

Another hypothesis considers parameatal cysts a subset of median raphe cysts, which may arise anywhere along the ventral midline from the meatus to the perineum. These may result from tissue trapping due to abnormal epithelial fusion during embryogenesis. However, this remains uncertain given differences in the histogenesis of the fossa navicularis [4].

Diagnosis is clinical, with pathology used for confirmation and classification. Differential diagnoses include epidermoid cyst, pilosebaceous cyst, fibroepithelial polyp, and juvenile xanthogranuloma [7]. Dermoscopy may assist in evaluation [14].

Histopathology commonly demonstrates columnar, cuboidal, squamous, or transitional epithelium. Otsuka et al. classified cysts into urethral (70.1%), epidermal (10.9%), and mixed (4.6%) types [15, 16]. Case 1 was a urothelial‐type cyst, while Case 2 represented a rare mixed‐type cyst.

Symptoms vary; while many patients present due to cosmetic concerns, others report discomfort, urinary stream changes, or dyspareunia. Trauma may cause bleeding, infection, or rarely spontaneous rupture [17]. No associations with race, sexual activity, or infection have been established, and the small number of reported cases limits epidemiologic analysis.

Treatment options include needle aspiration, marsupialization, and complete excision. Complete excision provides superior cosmetic and recurrence outcomes. Recurrence is common after aspiration or marsupialization, and spontaneous resolution occurs in 6%–25% of cases. Total excision with meatal reconstruction remains the preferred treatment [4, 8]. Both cases here underwent complete excision under sedation with excellent outcomes and no recurrence to date.

## 4. Conclusion

Parameatal urethral cysts are rare benign lesions usually presenting in childhood. Their etiology remains unclear, and epithelial composition varies. Although many cases are asymptomatic, some patients experience urinary or pain‐related symptoms. These two cases add to the limited literature and further support complete surgical excision as a safe and effective treatment with excellent cosmetic and functional outcomes.

## Funding

No funding was received for this manuscript.

## Consent

No written consent has been obtained from the patients as there is no patient‐identifiable data included in this case report/series.

## Conflicts of Interest

The authors declare no conflicts of interest.

## Data Availability

The data that support the findings of this study are available on request from the corresponding author. The data are not publicly available due to privacy or ethical restrictions.
